# Chitosan-Dextran sulfate coated doxorubicin loaded PLGA-PVA-nanoparticles caused apoptosis in doxorubicin resistance breast cancer cells through induction of DNA damage

**DOI:** 10.1038/s41598-017-02134-z

**Published:** 2017-05-19

**Authors:** Sumit Siddharth, Anmada Nayak, Deepika Nayak, Birendra Kumar Bindhani, Chanakya Nath Kundu

**Affiliations:** 1 Cancer Biology Division, KIIT School of Biotechnology, KIIT University, Campus-11, Patia, Bhubaneswar, Odisha 751024 India; 2 Plant Biotechnology and Nanotechnology Division, KIIT University, Campus-11, Patia, Bhubaneswar, Odisha 751024 India

## Abstract

To overcome the toxicity, pharmacokinetics and drug resistance associated with doxorubicin (DOX), a strategy was developed by encapsulating DOX- loaded-PLGA-PVA- nanoparticles within chitosan-dextran sulfate nanoparticles (CS-DS) [CS-DS-coated-DOX-loaded -PLGA-PVA-NP] and study the sensitivity against DOX- resistance- breast cancer cells (MCF-7-DOX-R). These CS-DS and PLGA-PVA double coated DOX are spherical, stable, polydispersed and have zeta potential +2.89 mV. MCF-7- DOX-R cells were derived by exposing increasing doses of DOX in MCF-7 cells. These cells were resistance to 500 nM of DOX while parental cells were susceptible at 150 nM. The double coated NP caused more cytotoxicity in cancer and MCF-7-DOX-R cells without affecting the normal cells in comparison to DOX-loaded-PLGA-PVA-NP. These NP enhances the uptake of DOX in MCF-7-DOX-R cells and caused apoptosis by increasing apoptotic nuclei, Bax/Bcl-xL ratio, cleaved product PARP-1, tumor suppressor gene p21, p53, topoisomerase inhibition activity, DNA damage and decreasing the migratory potential of cells. An increased S phase arrest was noted in DOX and DOX- loaded- PLGA-PVA-NP treated cells but reduction of S phase and simultaneous increase of Sub-G1 was observed in double coated-NP. Thus, data revealed that CS-DS- DOX- loaded PLGA-PVA- NP caused DOX-resistance cell death by inducing inhibition of topoisomerase activity followed by DNA damage.

## Introduction

Doxorubicin (DOX) belonging to anthracycline family is an age old antibiotic and anti neoplastic drug widely used in the treatment of cancer. As a mechanism of action it intercalates into the DNA thus inhibiting macromolecular synthesis. The drawbacks associated with DOX based chemotherapy is that; it affects healthy cells apart from cancer cells, cancer cells develop DOX resistance and sometimes DOX causes biventricular failure leading to cell death. These drawbacks of cardiotoxicity, drug resistance and normal cell damage associated with DOX are the major hindrances for its efficiency against breast cancer which limits its clinical use and demands the development of new formulation of drug^1^.

Cancer cells exhibits resistance mechanism to chemotherapeutic drugs due to one of the following mechanism i.e. enhanced detoxification of the drugs through increased metabolism and decrease in drug uptake. Thus development of agents that overcome the drug efflux and resistance with high efficiency and low toxicity has been the focus of wide research^2^.

Nanotechnology holds good to overcome drug resistance by means of targeted delivery and gained more attention due to unique accumulation behavior. Similarly, to overcome drug resistance and decrease the side effects of doxorubicin, nanotechnology holds promising potential by employing targeted drug delivery approach. Past 2–3 decades have seen rigorous research on nanomedicine for cancer treatment. Nanocarriers, such as hydrogels, polymeric nanoparticles, liposomes, and self-assembling nanofibers enhances the therapeutic efficiency of anticancer drugs by facilitating local drug uptake and developing drug bioavailability due to the passive targeting ability by the enhanced permeability and retention (EPR) effect^3^. It has been reported that association of DOX with liposome significantly reduced the dose dependant cardiac toxicity^4^. However, very little work has been carried out for targeting DOX resistant breast cancer utilizing DOX nanoparticles.

Chitsoan is a biocompatible, biodegradable cationic polymer possessing mucoadhesive properties. It exhibit low toxicity and enhances the penetrating potential of molecules across mucosal surfaces^5^.

On these premises, our idea here was to develop an experimental strategy for encapsulation of DOX loaded PLGA-PVA nanoparticles within chitosan-dextran sulfate nanoparticles. We hypothesized to perform a dual coating on DOX with PLGA-PVA and CS-DS nanoparticles to enhance the effectiveness of DOX, to overcome DOX resistance and to reduce the toxicity associated with the same.

## Results

### Synthesis and characterization of DOX loaded PLGA-PVA nanoparticles and CS-DS coated DOX loaded PLGA-PVA nanoparticles

**Figure 1:**
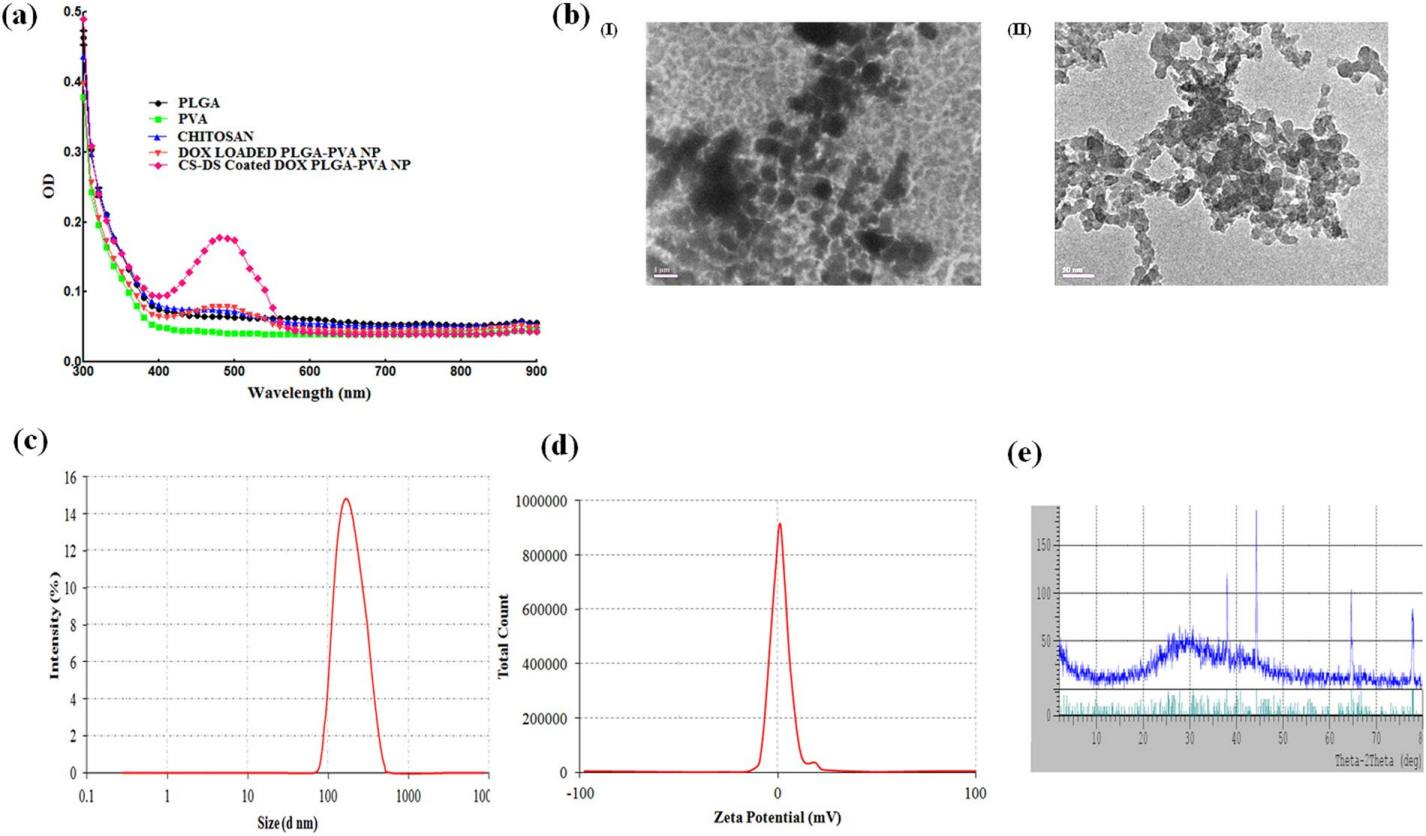
Characterization of DOX nanoparticles. (**a**) UV-Vis spectral analysis of PLGA, PVA, Chitosan, DOX loaded PLGA-PVA NP and CS-DS coated DOX loaded PLGA-PVA NP. (**b**) (I) and (II) DOX loaded PLGA-PVA NP and CS-DS coated DOX loaded PLGA-PVA -NP size and shape analysis by TEM, respectively. (**c**) Size distribution analysis of CS-DS coated DOX loaded PLGA-PVA NP. (**d**) Zeta potential analysis showing surface charge distribution of CS-DS coated DOX loaded PLGA-PVA NP. (**e**) XRD pattern of CS-DS coated DOX loaded PLGA-PVA NP. Images are representative of three different experiments.

CS-DS coated DOX loaded-PLGA-PVA-NP showed high degree of stability indicated by UV-Vis spectrophotometric analysis (Fig. 1a). A characteristic peak at 480 nm by DOX loaded- PLGA-PVA and CS-DS coated DOX loaded-PLGA-PVA-NPs was noted (Fig. 1a). Interestingly, highest peak was shown by CS-DS coated DOX loaded PLGA-PVA-NPs (Fig. 1a). It was also observed that the nanoparticles did not form any precipitation or aggregation upto 120 days of storage which indicates that the nanoparticles are very stable. TEM data revealed that DOX loaded PLGA-PVA as well as CS-DS coated DOX loaded PLGA-PVA-NPs are spherical and polydispersed with the size of 1 µm and 50 nm, respectively (Fig. 1b I & II). DLS analysis showed that formulated CS-DS coated DOX loaded PLGA-PVA-NP had an average diameter 178.2 ± 2.5 d.nm (Fig. 1c). The zeta potential or net surface charge of the NP is +2.98 ±0.32 mV (Fig. 1d). Figures 1e demonstrate nearly face centered cubic structure (FCC) of the formulated CS-DS-DOX –PLGA-PVA-NPs (Fig. 1e).

### CS-DS coated DOX loaded PLGA-PVA-NP is more cytotoxic in DOX resistant breast cancer cells

**Figure 2:**
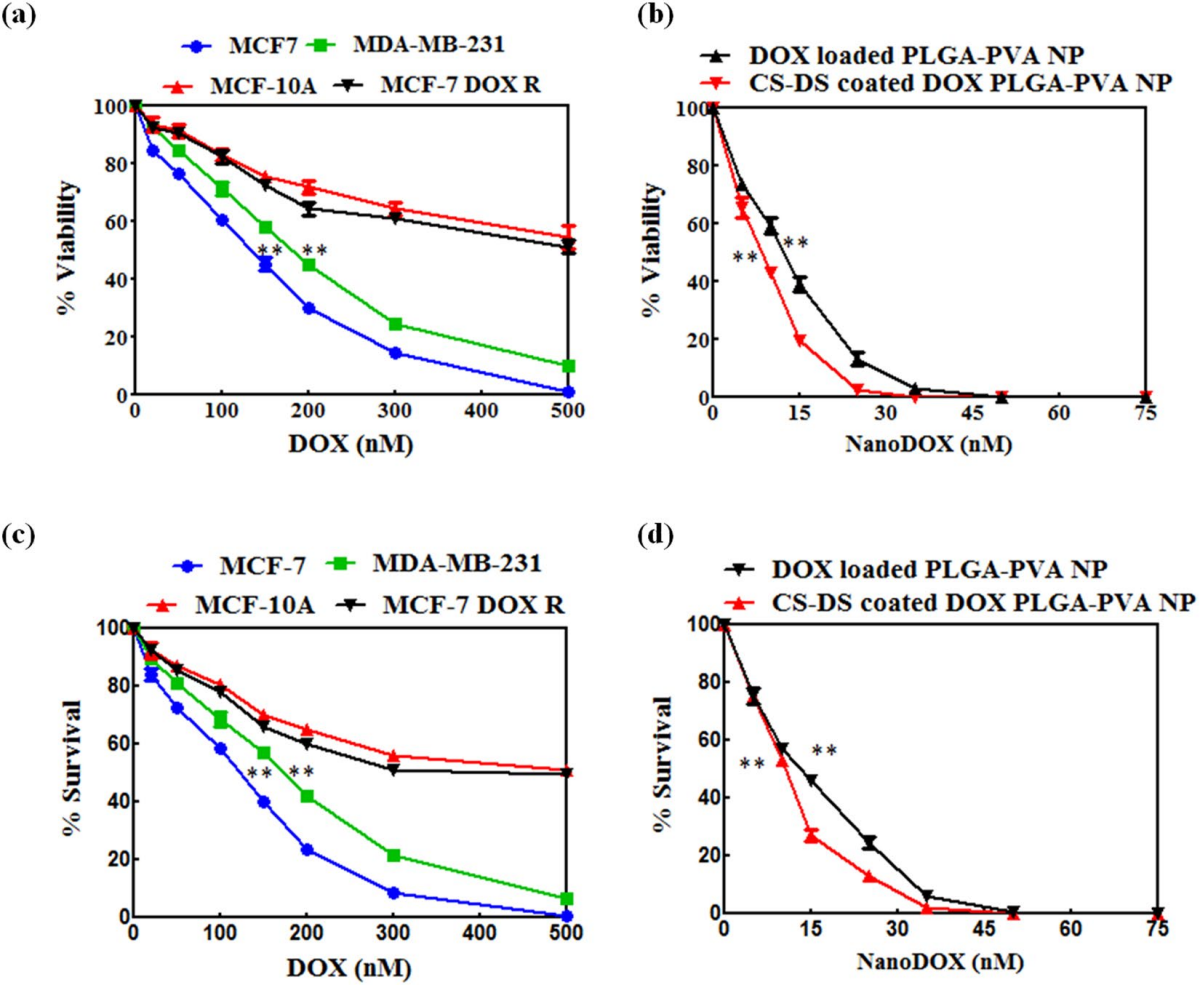
CS-DS coated DOX- loaded- PLGA-PVA- NP is more cytotoxic to DOX resistant breast cancer cells. (**A**) MTT cell viability assay of DOX treated different breast cancer cells (MCF-7 and MDA-MB-231), normal breast epithelial cells (MCF-10A) and DOX resistant breast cancer cells (MCF-7-DOX-R). (**B**) MTT cell viability assay of DOX loaded PLGA-PVA NP and CS-DS coated DOX loaded PLGA-PVA NP treated MCF-7 DOX-R cells. (**C**) Clonogenic cell survival assay of DOX treated different breast cancer cells (MCF-7 and MDA-MB-231), normal breast epithelial cells (MCF-10A) and MCF-7- DOX-R cells. (**D**) Clonogenic cell survival assay of DOX -loaded PLGA-PVA NP and CS-DS coated DOX loaded PLGA-PVA NP treated MCF-7- DOX-R cells. Data include mean ± S.E.M. of three different experiments. Statistical significance was determined by one way ANOVA followed by Bonferroni multiple comparision test; **p < 0.0005.

To measure the anti- cell proliferative efficacy of DOX, DOX loaded PLGA-PVA nanoparticles and CS-DS coated DOX loaded PLGA-PVA-NPs, an MTT cell viability assay was carried out as described in materials and methods. First, a DOX resistance MCF-7 cell lines (MCF-7 DOX-R) was developed by the procedure described in material and methods. Data revealed that DOX caused a dose dependant decrease of cell viability in MCF-7 and MDA-MB-231 cells with an IC_50_ (inhibition of fifty percent cell growth in culture) of 150 nM and 200 nM, respectively. Interestingly, no IC_50_ reached in MCF-10A and DOX resistant MCF-7 (MCF-7 DOX-R) cells till 500 nM treatment of DOX (Fig. 2a). Figure 2b shows the anti-proliferative effects of DOX loaded PLGA-PVA nanoparticles and CS-DS coated DOX loaded PLGA-PVA NPs on MCF-7- DOX-R cells. Our results illustrate both DOX loaded PLGA-PVA -NPs and CS-DS coated DOX loaded PLGA-PVA-NPs caused a decrease of cell viability of DOX resistant MCF-7 (MCF-7-DOX-R) cells with an IC_50_ of 15 nM and 8 nM, respectively. This data clearly demonstrate that CS-DS coated DOX loaded PLGA-PVA-NPs are more effective against MCF-7-DOX-R cells compared to DOX loaded PLGA-PVA-NPs.

Next, we wanted to study the effect of above agents on the colony forming ability of MCF-7, MDA-MB-231 and MCF-7- DOX-R breast cancer cells along with normal breast epithelial cells (MCF-10A) (Fig. 2c and d). Figure 2c demonstrates that DOX inhibits the colony forming ability of MCF-7 and MDA-MB-231 cells with an LC_50_ of 150 nM and 200 nM, respectively. However, no LC_50_ reached till 500 nM treatment in MCF-7-DOX-R and MCF-10A cells, respectively. Both DOX loaded PLGA-PVA -NPs and CS-DS coated DOX loaded PLGA-PVA- NPs significantly inhibited the colony forming ability of MCF-7- DOX-R cells with an LC_50_ of 15 nM and 8 nM, respectively (Fig. 2d). Thus data suggested that CS-DS coated DOX loaded PLGA-PVA-NP is more cytotoxic in DOX resistance cells compare to only DOX loaded NP.

### CS-DS coated DOX loaded PLGA-PVA-NP inhibits topoisomerase activity and cell migration

**Figure 3:**
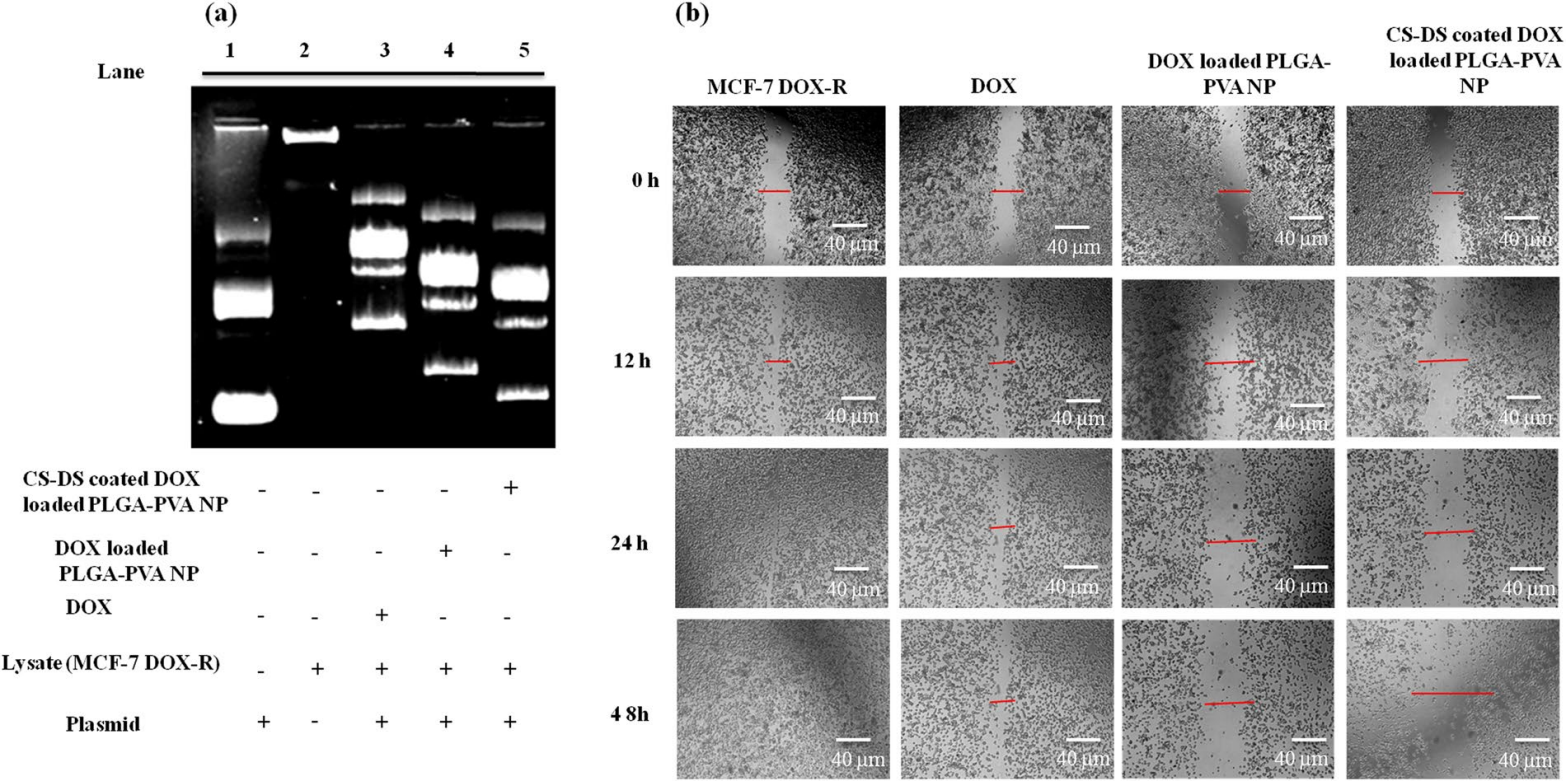
CS-DS coated DOX loaded PLGA-PVA -NP inhibits topoisomerase activity and retards cell migration of MCF-7- DOX-R cells. (**a**) Inhibition of topoisomerase activity by DOX nanoparticles. (**b**) Effect of DOX nanoparticles on cell migration of MCF-7- DOX-R cells. Images shown here were the representatives of three independent experiments.

It is a well known fact that DOX inhibits topoisomerase enzyme activity and inhibition of topoisomerase activity is one of the hallmark properties of any anti-cancer agent^6^. So, topoisomerase inhibition activity of the nano formulated DOX was evaluated using a plasmid based in gel assay system according to protocol described earlier^6^. The DOX resistance cells (MCF-7-DOX-R) were treated with NPs with fixed concentration of NP (CS-DS –coated DOX-loaded PLGA-PVA-NP -8 nM and DOX loaded PLGA-PVA-NP −15 nM) for 48 h and experiment was carried out according to protocol described above. In untreated lysate, the supercoiled plasmid was completely relaxed or linear which fails to migrate into the well (Fig. 3a, lane 2). This is only possible if the topoisomerase activity of the nuclear lysate of MCF-7- DOX-R cells remain intact. Contrastingly, the topology of supercoiled DNA was observed upon respective drug treatment with highest supercoiling observed in CS-DS coated DOX loaded PLGA-PVA-NPs (Fig. 3a lane 5) followed by DOX loaded PLGA-PVA-NP (Fig. 3a, lane 4). The migration of DNA was seen in lane 1 for plasmid only. Next, we wanted to study the anti-cell migratory effect of various forms of DOX-NP on MCF-7- DOX-R cells. This was done by a well established wound healing assay (Fig. 3b). In untreated cells, migration occurred and the wound healed within 48 h. However, the cell migration significantly inhibited upon various treatments, with maximum inhibition observed in CS-DS coated DOX loaded PLGA-PVA-NP treatment followed by DOX loaded PLGA-PVA-NP (Fig. 3b). DOX treated MCF-7- DOX-R cells revealed a non-significant decrease of cell migration.

### CS-DS coated DOX loaded PLGA-PVA-NP enhances the cellular uptake of DOX in MCF-7-DOX-R cells and induces apoptosis

**Figure 4:**
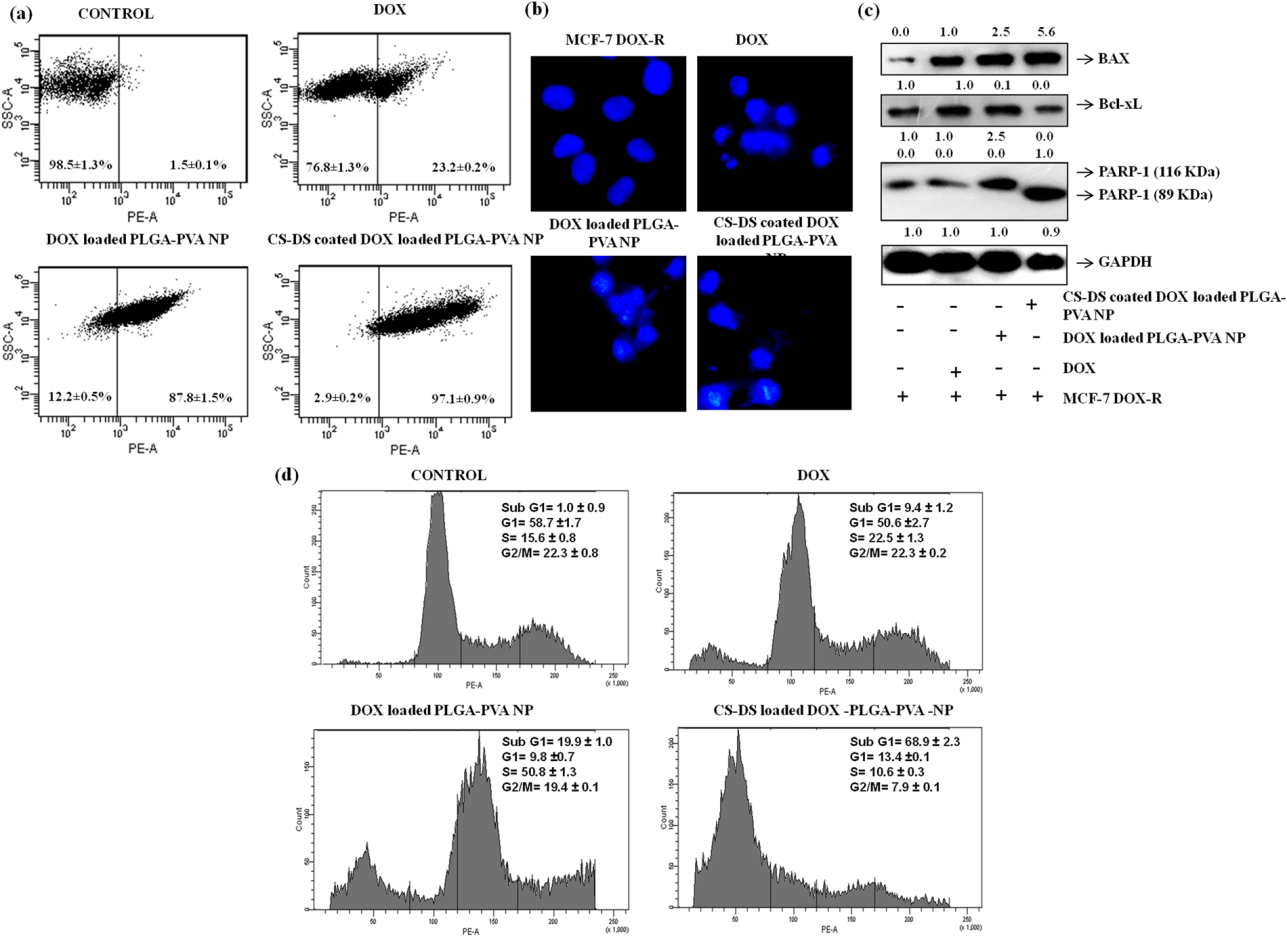
CS-DS coating on DOX loaded PLGA-PVA- NP enhances the uptake of DOX and induces apoptosis in MCF-7- DOX-R cells. (**a**) Cellular uptake of DOX and DOX -NP in MCF-7-DOX-R cells measured by FACS. Data represents mean ± S.E.M. of three different experiments. (**b**) Apoptotic nuclei of DOX and DOX- NP treated MCF-7- DOX-R cells after DAPI staining. Images are representative of three independent experiments. (**c**) Western blot analysis of BAX, Bcl-Xl and PARP-1 in drug treated cellular lysate of MCF-7- DOX-R cells. Data was the one of the replica of three different experiments. (**d**) Regulation of cell cycle profile after treatment with DOX and DOX NP in MCF-7- DOX-R cells. Data was the one of the replica of three different experiments.

The cellular uptake of DOX increased upon nano-formulation and maximum cellular uptake (97.1%) was noted with CS-DS coated DOX loaded PLGA-PVA-NP followed by PLGA-PVA capped DOX (87.8%) compared to uncoated DOX (23.2%) (Fig. 4a). To analyze the apoptotic effects of DOX, DOX loaded PLGA-PVA-NP and CS-DS coated DOX loaded PLGA-PVA-NP in MCF-7- DOX-R cells, a series of experiments were carried out. At first apoptotic nuclei were measured after staining with DAPI nuclei binding dye in agents treated cells. Figure 4b revealed highest number of fragmented, shrunken and bubble shaped nuclei in CS-DS coated DOX loaded PLGA-PVA-NP treated MCF-7- DOX-R cells followed by DOX loaded PLGA-PVA-NP and DOX treated MCF-7-DOX-R cells, respectively (Fig. 4b). Next, changes of representative apoptotic markers were measured using western blots. Increased BAX/Bcl-XL ratio was noted with a maximum of 10 fold induced BAX and 2 fold reduced Bcl-XL in CS-DS coated DOX loaded PLGA-PVA-NP treated MCF-7- DOX-R cells. However, a non-significant change of BAX/BcL-XL ratio was noted in DOX loaded PLGA-PVA-NP and DOX treated cells, respectively. We measured the expression of another apoptotic marker, PARP-1 (116 KDa) and the respective cleaved product of PARP-1 (89 KDa) in treated MCF-7 DOX-R cells. Data revealed that the cleaved product of PARP-1 appeared only upon CS-DS coated DOX loaded PLGA-PVA-NP treated resistant cells with a complete abolishment of full length of PARP-1 (Fig. 4c) compared to DOX loaded PLGA-PVA-NP and DOX treated resistant cells, respectively. To further confirm the apoptosis a FACS analysis was carried out in PI stained of treated cells. More than 68 percent apoptotic cells were noted in sub G1 phase in CS-DS coated DOX loaded PLGA-PVA-NP treated cells which is more than 50 percent than DOX loaded PLGA-PVA-NP treated cells (Fig. 4d). A significant increased of S phase arrest of cells were noted in DOX loaded PLGA-PVA-NP in compare to DOX alone. Interestingly, after coated with CS-DS of DOX loaded PLGA-PVA-NP cells, a decrease S phase arrest and simultaneous increased apoptosis was noted (Fig. 4d). Thus, data appears that after coating with CS-DS of NP, cells were moved from S phase to apoptosis.

### CS-DS coated DOX loaded PLGA-PVA- NP increases the cell sensitivity by inducing DNA damage

**Figure 5:**
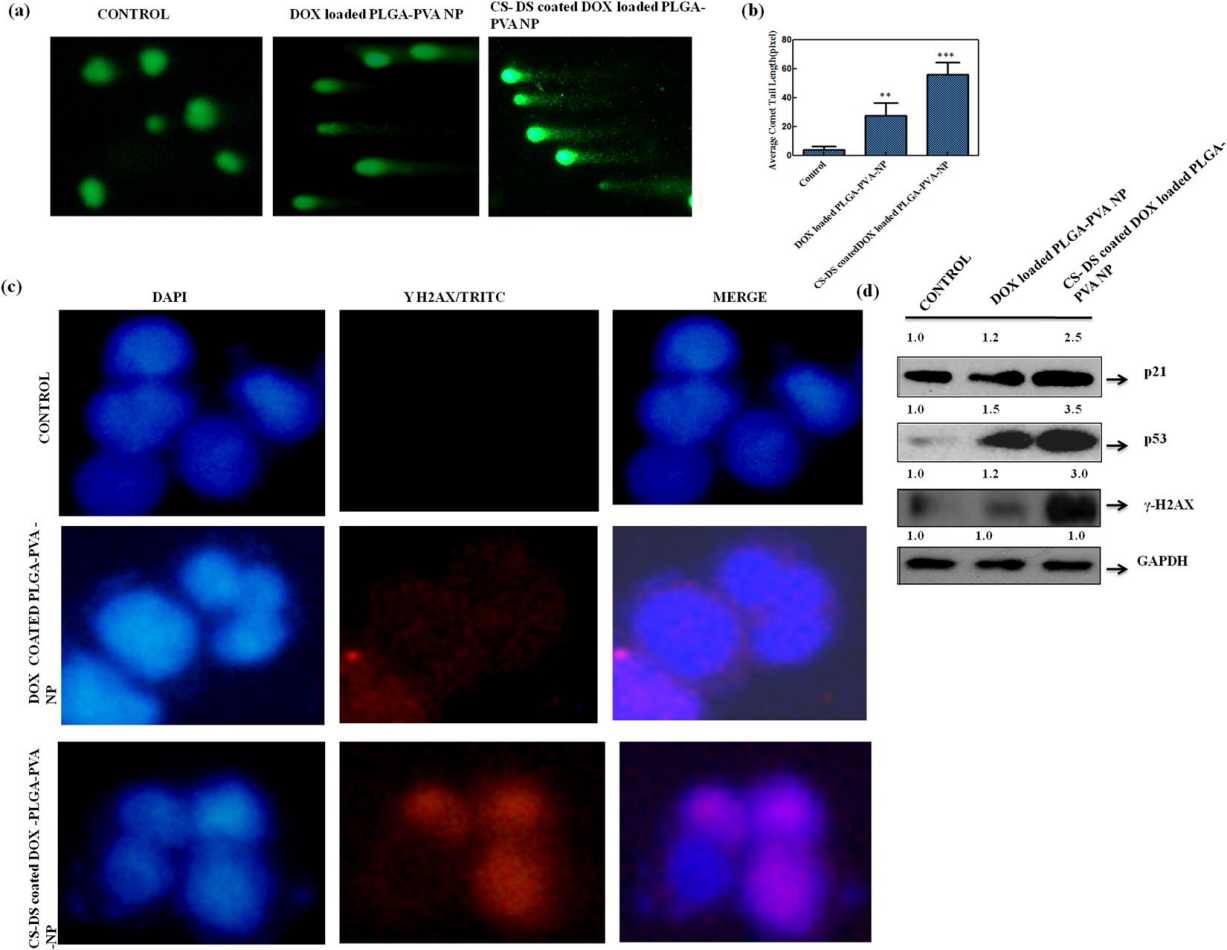
CS-DS coating on DOX loaded PLGA-PVA-NP induces DNA damage in MCF-7- DOX-R cells. **(a)** DNA damage (comet formation) increased after treatment with NPs. (**b**) Histogram representing average comet length in pixel. (**c**) Immunocytochemistry images of γ H2AX expressions in cells after treatment with NPs. Cells were stained with γH2AX and nuclei were stained with DAPI and images were taken under fluorescence microscope (Nikon, Japan). (**d**) Expression of representative proteins involved in DNA damage.

Next, we wanted to study the underlying mechanism of apoptosis caused by NP. NP inhibits the topoisomerase inhibition activity in MCF-7-DOX-R cells and arrests the cells at S phase or translocates from S phase to apoptosis. It is a well documented fact that replication is taken place in S phase. Thus, data from above experiment suggests NP mediated apoptosis might involved DNA damage process. To check the hypothesis we have measured the DNA damaging efficacy of NP treated cells using two (Comet and γH2AX) well established cell based assays according to protocol described in methods and materials. Figure 5a showed an increased of comet formation and comet length in NP treated cells in comparison to untreated one. But the higher comet formation and increase comet length (2.5 fold) was noted in CS-DS coated DOX loaded PLGA-PVA-NP than DOX-loaded PLGA-PVA-NP treated cells (Fig. 5a). Figure 5b is the graphical representation of the average comet length indicating maximum comet length formation in CS-DS coated NP (Fig. 5b). Next, we have checked the γH2AX expression after staining the cells with antibody. Maximum increased of γH2AX expression was noted in CS-DS coated DOX loaded- PLGA-PVA-NP than either DOX loaded PLGA-PVA-NP or untreated cells (Fig. 5c). Then, we measured the expression of tumor suppressor gene (p21, p53) and γH2AX in western blot. Figure 5d showed increased protein expressions of p21, p53 and γH2AX in NP in compare to untreated cells but maximum expression was noted in CS-DS coated DOX loaded PLGA-PVA-NP treated cells.

## Discussion

Although DOX is known to be a well known anti-cancer agent but it has serious drawbacks due to toxicity in normal cells, low bioavailability, frequently got resistance as well as need higher doses for reaching optimal therapeutic response. To eliminate the toxicity, increased the bioavailability, stability and to make more sensitive to cancer cells in the current study, we have designed two different types of DOX loaded nanoparticles and studied their efficiency in DOX resistant breast cancer cells as a model system. To fulfill our objective, we have first established a stable DOX resistance breast cancer cell line (MCF-7- DOX-R) by exposing MCF-7 cells to DOX by the procedure described earlier^7^. Cells were initially grown in low dose of DOX and then slowly increased the dose upto 500 nM which is far above than normal therapeutic concentration. We characterized the cells as MCF-7-DOX-R cells. We found that the MCF-7-DOX-R cells were resistance to DOX till 500 nM irrespective of the fact that the non-resistant MCF-7 was susceptible to DOX at much lower concentration (150 nM).

For the synthesis of DOX loaded PLGA-PVA nanoparticles, we capped DOX with PLGA-PVA and formulated DOX loaded PLGA-PVA nanoparticles. Secondly, we prepared the nanoparticles of chitosan and dextran sulfate and termed them as CS-DS nanoparticles. Then, we coated the DOX loaded PLGA-PVA nanoparticles with CS-DS nanoparticles which reduced the size of from 1 µm (DOX loaded PLGA-PVA-NP) to 50 nm (CS-DS coated DOX loaded PLGA-PVA-NP). Coating of DOX loaded PLGA-PVA-NPs with CS-DS nanoparticles enhanced DOX stability, absorption, bioavailability, and uptake by DOX resistance breast cancer cells (Fig. 4a). Because these nanoparticles have been designed and formulated to target specific tumor tissue and cells, hence, they must possess a long shelf life in the circulatory system to deliver pharmacologic agents to the tumor cells. We found that CS-DS coating on DOX loaded PLGA-PVA-NP enhanced the topoisomerse inhibitory and anti-cell migratory potential of DOX (Fig. 3). The CS-DS coated DOX loaded PLGA-PVA-NP caused apoptosis in cancer cells and DOX resistance cancer cells by minimally affecting normal epithelial cells. It is interesting to note that double coating of the DOX is more effective to kill the cells than single coating DOX as evident from cell apoptotic data (cell cycle, DAPI staining nuclei and western data). Enhanced Bax/Bcl-XL, PARP-1 cleavage, and more fragmented, bubble shape, shrinkage nuclei and also fifty percent more cell death was noted in CS-DS coated DOX loaded PLGA-PVA-NP in comparison to DOX loaded PLGA-PVA-NP (Fig. 4).

**Figure 6:**
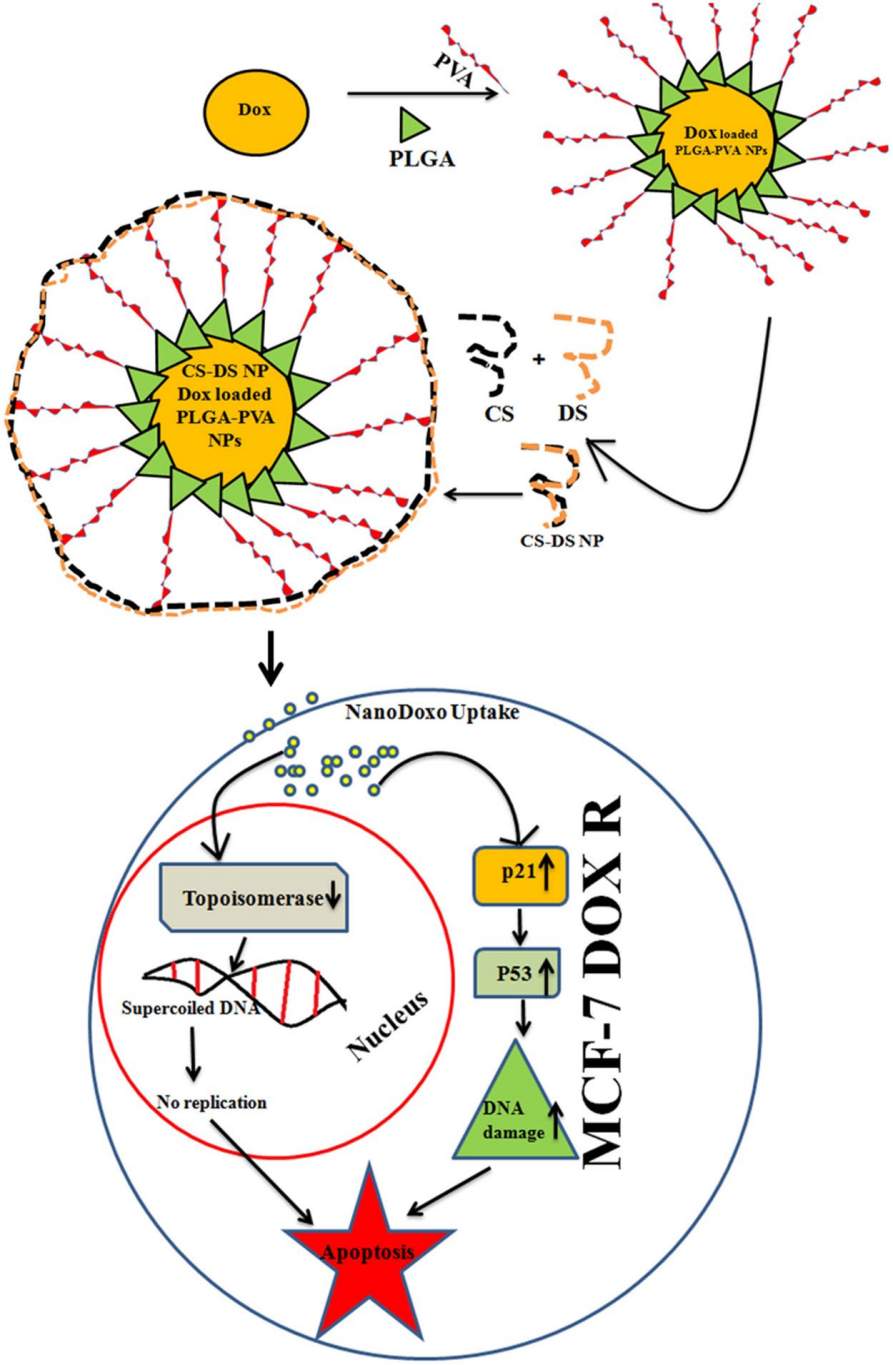
Graphical representation showing the formation of CS–DS coated DOX loaded PLGA-PVA NP and its apoptotic activity in MCF-7- DOX-R cells. NP enters into the nucleus and inhibits the topoisomerase activity. Inhibition of topoisomerase activity increases the DNA damage and ultimately leads to cell death.

Next, we study the underlying mechanism of apoptotic efficacy of NP in DOX resistance cells. Cell cycle analysis showed that DOX alone can arrest the cells 22 percent at S phase which is 7 percent higher than control. But approximately 50 percent S phase arrest was noted in presence of DOX loaded PLGA-PVA-NP. Interestingly, a sharp drop of S phase arrest and simultaneous increased apoptosis was noted in presence of CS-DS-DOX loaded PLGA-PVA-NP (Fig. 4). It is also noted that NP inhibits the topoisomerase activity in cells (Fig. 3). These observations suggest that NP disrupts the regulation of cell cycle in S phase and cells are unable to cross the S-G2 transition. The observation suggest there might be some problem in S phase during the replication and might also be excessive DNA damage was the one of the major cause for cell death. In agreement with above observation and increase comet formation and comet length, γH2AX expression and increased level of S-G2 transition regulating protein (p21,p53) confirm NP mediated apoptotsis in DOX resistance cells is mediaed through induction of DNA damage. Finally, we have provided a flow diagram of the NP mediated DOX resistance breast cancer cell death (Fig. 6).

In conclusion, our study provides a new strategy of dual coating of DOX which facilitates the uptake of DOX by leading to topoisomerase inhibition and causing DNA damage thereby inducing apoptosis of the DOX resistant breast cancer cells. We advocate that CS-DS coated DOX loaded PLGA-PVA NP has great potential against DOX resistant breast cancer cells. The data and the promises shown by the CS-DS coated DOX loaded PLGA-PVA nanoparticles in our preclinical study indicate the high efficiency of this formulation for further translation into clinics.

## Materials and Methods

### Synthesis of DOX loaded PLGA-PVAnanoparticles

DOX loaded PLGA-PVA- nanoparticles were prepared following the protocol mentioned earlier^8^ with minor modifications. Briefly, 90 mg of PLGA was dissolved in 10 ml of acetone for 3 h on a magnetic stirrer at 800 rpm. Then, 20 mL of aqueous solution containing 2% PVA (w/v) (M.W. 30, 000–70,000) was prepared.1 mg of DOX was added to it and kept on a magnetic stirrer for 3 h at 800 rpm to get a uniform PVA-DOX solution. The organic solution of PLGA was added drop wise to the aqueous solution under continuous stirring at 800 rpm. A precipitation was observed in the aqueous layer and the suspension was stirred for 24 h to completely evaporate acetone. Un entrapped DOX was removed by centrifugation at 5000 rpm for 15 minutes and the DOX loaded PLGA-PVA nanoparticles were recovered by ultra-centrifugation at 35000 rpm for 20 minutes at 4 °C followed by overnight lyophilization (Free Zone Bench top Freeze Dry System, Lanconco, MO, USA). 1 mg/ml aqueous solution of DOX loaded PLGA-PVA nanoparticles were used for further experimentation.

### Synthesis of Chitosan-Dextran sulfate (CS-DS) nanoparticles

2 mg/mL chitosan solution was prepared in 0.1% acetic acid on magnetic stirring at 800 rpm. Similarly, 0.5 mg/mL dextran sulfate was prepared in deionized water. CS-DS nanoparticles were prepared by adding equal volume of CS solution in DS solution on a constant stirring at 800 rpm overnight.

### Synthesis of CS-DS coated DOX loaded PLGA-PVA nanoparticles

DOX loaded PLGA-PVA nanoparticles (1 mg/mL) were mixed in CS-DS solution in 2:5 ratio and incubated on a magnetic stirrer at 800 rpm overnight. Free CS-DS particles were removed by centrifugation at 5000 rpm for 15 minutes. The CS-DS coated DOX loaded PLGA-PVA nanoparticles were collected by ultra-centrifugation at 35000 rpm for 20 minutes at 4 °C.

### Characterization of CS-DS coated DOX loaded PLGA-PVA nanoparticles

#### UV–Vis spectra analysis

The absorption spectra of the DOX nanoformulation were measured by EPOCH multivolume spectrophotometer (BioTek Instruments, USA) in regular intervals to determine the stability and quality of the NPs following the protocol mentioned earlier^9^. After the formulation of DOX loaded PLGA-PVA- NP and CS-DS coated DOX loaded PLGA-PVA- NP, a spectral scan analysis was performed in the range of 300–900 nm wavelength. We continued observing the spectral analysis for 30 days after every 24 h. The characteristic peaks obtained for PLGA, PVA, Chitsoan, DOX loaded PLGA-PVA- NP and CS-DS coated DOX loaded PLGA-PVA- NP were plotted and compared to conclude the uniqueness of the formulation.

### Transmission Electron Microscopy (TEM)

Nanoformulations of DOX were evaluated for size using TEM (JEOL-JEM 2100, Japan). NP (0.5 mg/mL) were suspended in water, sonicated for 30 s followed by adding one drop of the suspension on the carbon films supported by copper grid. Images were visualized at 120 kV under the microscope.

### XRD analysis

DOX loaded PLGA-PVA and CS-DS coated DOX loaded PLGA-PVA- NP were subjected to XRD analysis to reveal the crystalize nature of nanoparticles following the protocol mentioned earlier^8^. In brief, lyophilized form of the DOX loaded PLGA-PVA and CS-DS coated DOX loaded PLGA-PVA- NP were coated on XRD grid and the spectra were recorded using powder X-ray diffractometer (D8 Advance Powder XRD, Bruker, USA). The diffracted intensities were recorded from 5 to 80° at 2θ angles.

### Particle size analysis and zeta-potential measurements

Dynamic light scattering (DLS) was used to measure the hydrodynamic diameter and laser Doppler anemometry was used to determine zeta-potential (mV) according to protocol described earlier^9^. The DLS and laser Doppler anemometry analyses were performed using a Zetasizer Nano ZS (Malvern Instruments, Worcestershire, UK). The DLS measurements were carried out at a wavelength of 532 nm at 25 °C with an angle detection of 90°.

### MTT cell viability assay

Anchorage dependant cell viability of cells after treatment with the agents were measured using MTT cell viability assay following the protocol mentioned earlier^8, 10^. In brief, 1 × 10^3^ exponentially growing cells were seeded in triplicate in 96 well flat bottom tissue culture plates for 80% confluence. Then, the cells were treated with varied concentrations of DOX, DOX-NP for 48 h. After the treatment, the media were discarded and the cells were washed once with 1X PBS and 0.05% MTT reagent was added to each well and incubated for 5 h at 37 °C in a 5% CO_2_ incubator for formazan crystal formation. The crystals were dissolved in 100 µL/well of 0.2% NP-40 detergent solution and the absorbance was read at 570 nm using microplate reader (Berthold, Germany). Data obtained were represented graphically as % viability versus concentrations^11^.

### Establishment of DOX resistant breast cancer cells (MCF-7 DOX-R)

To establish DOX resistant MCF-7 cells, we followed a previously described protocol^7^. Initially, MCF-7 cells were grown in cell culture media containing 10 nM DOX for 70% confluence. Once the cells attained the confluency, the cells were trypsinized and further exposed to 20 nM of DOX to attain 70% confluence. We continued this process for stepwise selection of resistant cell still a final concentration of 500 nM.

### Clonogenic cell survival assay

The colony forming ability of breast cancer cells along with normal breast epithelial cells upon DOX, DOX-NP exposure was measured by clonogenic cell survival assay using a well defined assay described earlier^9^. Cells (500 cells/ well) were treated with varied concentrations of DOX and DOX -NP for 48 h. Then, the media were replaced with fresh media and the cells were allowed to grow for 7–8 doublings. Then, the cells were washed with 1X PBS and followed by staining with 0.2% crystal violet for 1 h. Stained colonies were counted microscopically using colony counter and the data were plotted graphically as percent survival against concentrations.

### Cell migration assay

Anti-cell migratory effect of DOX and DOX-NP was studied by a wound healing assay following the previously mentioned protocol^6^. In brief, MCF-7-DOX-R cells were grown in 35 mm cell culture discs to 90% confluence, following which a wound is created across the center in the cell monolayer by a sterile microtip. The cells were washed with fresh medium to remove the cell debris. Then, the cells were treated with DOX and DOX -NP and were allowed to migrate in the medium. The wound was assessed microscopically at different time intervals.

### Measurement of apoptosis by DAPI staining

To analyze the apoptotic cells with condensed and fragmented nuclei, DAPI nuclear staining was performed following the protocol mentioned earlier^6, 11^. Briefly, 1 × 10^6^ MCF-7-DOX-R cells were seeded in 60 mm cell culture discs and grown until 80% confluence. Cells were then treated with DOX and DOX-NPs 48 h. The cells were fixed with chilled acetone: methanol (1:1 ratio) for 20 min at 4 °C and then washed twice with 1X PBS. The cells were stained with DAPI (10 ng/mL) for 30 min at 37 °C and the nuclei were observed under fluorescence microscope (Nikon, Japan) at 40 x magnification.

### Comet assay

To check the DNA damaging efficacy of the formulated nanoparticles, comet assay was performed as per the protocol described earlier^12^. Cells were suspended in PBS and about 5000-6000 cells were mixed with low melting agarose at 37 °C and spread on a microscopic slide. After solidifying, the slides were dipped in chilled alkaline lysis for 1 h followed by incubation in alkaline electrophoresis buffer. Then electrophoresis was performed at 20 V for 20 min. Slides were then dipped in neutralization buffer (0.4 mol/L Tris-HCl and pH 7.5) and sequentially washed with distilled water, 70% ethanol then allowed to dry. Later, 40 μL of SYBR was added to each slide and after incubation in the dark for 30 min at RT images were captured using fluorescence microscope (Nikon, Japan). TriTek CometScore™ software was used to analyze the come length.

### Uptake of DOX, DOX loaded PLGA-PVA nanoparticles and CS-DS coated DOX loaded PLGA-PVA nanoparticles

Uptake analysis of DOX, DOX loaded PLGA-PVA nanoparticles and CS-DS coated DOX loaded PLGA-PVA-NPs were measured by flow cytometry as described earlier^8^ by using the DOX auto fluorescence property. In brief, 1 × 10^6^ MCF-7-DOX-R cells were seeded in 60 mm cell culture discs and incubated for 24 h. Then, the cells were exposed with agents for 3 h. Then, the media were discarded and the cells were washed with 1X PBS. The cells were then harvested by trypsinization and processed for uptake study by flow cytometer (FACS Canto II, Becton & Dickinson, CA, USA). PE filter was used for fluorescence detection and 10,000 gated populations were analyzed for each sample.

### Western blot analysis

To detect the expression of different proteins upon DOX and DOX- NP treatment on MCF-7-DOX-R cells, a western blot analysis was performed as described earlier^11^. Approximately, 5 × 10^5^ cells were treated with DOX and DOX-NPs for 48 h. The cells were then harvested and cellular lysates were prepared using modified RIPA lysis buffer. 40 µg of protein were loaded, separated on a 10% SDS-PAGE, transferred onto PVDF membrane and processed for western blotting following antibody specific manufacturer’s protocol. Numerical value above each panel of protein represents the band intensity calculated by UVP GelDoc-It ® 310 Imaging system (UVP, Cambridge, UK).

### Topoisomerase inhibition assay

Topoisomerases are nuclear proteins responsible for relaxing the supercoiled DNA. To study the topoisomerase inhibitory role of DOX, DOX -NPs, a topoisomerase inhibition assay was carried out following the previously described protocol^6^. Cells were grown in 60 mm cell culture dishes to 80% confluence and then treated with the agents for 48 h. Then, the cells were harvested and nuclear lysates were prepared using the protocol mentioned earlier^6^. The reaction mixture for topoisomerase contained 100 µg of nuclear protein, PGL2 p21 plasmid as substrate in a reaction buffer (200 mM Tris-Cl, pH-7.5, 920 100 mM MgCl2, 10 mM ATP, 10 mM EDTA, 10 mM dithiothreitol, 1.5 mM KCl, 300 μg/mL BSA) were incubated at 37 °C for 30 min. The reaction was stopped by adding 1% SDS. 30 µL of the reaction mixture was loaded in each well of 0.9% agarose gel electrophoresis containing EtBr. Electrophoresis was performed for 4 h at 15 V and photographs were taken using gel documentation system (UVP, Germany).

### Analysis of apoptosis and cell cycle regulation by FACS

In order to check apoptosis and regulation of cell cycle profile after treatment with agents, a FACS analysis was carried out as described earlier^8^. Briefly, 1 × 10^5^ cells were seeded in 6 well tissue culture plates and were treated with the above mentioned compound for 48 h. Cells were harvested by trypsinization and fixed with ice cold 70% ethanol and incubated over night at −20 °C and stained with Propidium Iodide (PI) containing 0.05% RNase prior to sample acquisition. Then cells were sorted using FACS with an event count of 10,000 events per sample. Analysis of data was done by FACS diva software.

### Immunocytochemistry of γH2AX expression

*In situ* γH2AX expression was carried out by immunocytochemistry as described earlier^12^. Cells were grown on coverslips and treated with NPs. Then cells were washed with 1X PBS and fixed with acetone:methanol in a 1:1 ratio for 20 min at 20 °C followed by blocking in 2% BSA and 0.02% triton X-100 in 1X PBS for 3 h at 4 °C. The cells were washed once with 1X PBS and incubated for 2 h at 4 °C. Unbound antibodies were removed by washing twice with 1X PBS. Secondary antibody conjugated to TRITC was added and the cells were incubated for 1 h at 4 °C. The cells were then washed thrice in 1X PBS and the nuclei were counter stained with DAPI to visualize nuclei. Images were captured under fluorescence microscope (Nikon, Tokyo, Japan) at 40X magnification

### Statistical analysis

The statistical significance was performed using Graph Pad Prism version 5 software, USA. The results were expressed as mean ± S.E.M. of 5different experiments. The data were analysed by one-way analysis of variance (ANOVA) followed by Bonferroni’s multiple comparison test. The statistical significance of difference in the central tendencies was designated as **p < 0.005 and ***p < 0.001.

## Electronic supplementary material

Supplementary Figures
