# Supplementary material for: Chitosan-Dextran sulfate coated doxorubicin loaded PLGA-PVA-nanoparticles caused apoptosis in doxorubicin resistance breast cancer cells through induction of DNA damage
Source: Sci Rep. 2017 May 19;7:2143. doi: 10.1038/s41598-017-02134-z (PMC5438340; doi:10.1038/s41598-017-02134-z)
Supplement: Supplementary file 1 — Supplementary Figures [file 41598_2017_2134_MOESM1_ESM.pdf]

Chitosan-Dextran sulfate coated doxorubicin loaded PLGA-PVA- nanoparticles caused apoptosis in doxorubicin resistance breast cancer cells through induction of DNA damage.

Sumit Siddharth[1], Anmada Nayak[1], Deepika Nayak[1], Birendra Kumar Bindhani[2*], Chanakya Nath Kundu[1*]

[1]Cancer Biology Division, KIIT School of Biotechnology, KIIT University, Campus-11, Patia, Bhubaneswar, Odisha, 751024, India.

[2] Plant Biotechnology and Nanotechnology Division, KIIT University, Campus-11, Patia, Bhubaneswar, Odisha, 751024, India.

Short title: Sensitivity of double coated DOX-NP against DOX resistance cancer cells.

To whom correspondence should be addressed: [*1]Chanakya Nath Kundu, Cancer Biology Division, KIIT School of Biotechnology, KIIT University, Campus-11, Patia, Bhubaneswar, Orissa, 751024, India. Tel. : +91-0674-272-5466; Fax: +91-0674-272-5732; E-mail: cnkundu@gmail.com and Birendra Kumar Bindhani[2*], Plant Biotechnology and Nanotechnology Division, KIIT University, Campus-11, Patia, Bhubaneswar, Odisha, 751024, India. Tel. : +91-0674-272-5466; Fax: +91-0674-272-5732; E-mail: drbindhani@gmail.com

Methods and material:

1) It is confirmed that all methods were carried out in accordance with relevant guidelines and regulations.

2) In current MS we did not use any animal or human subject so Institutional ethical approval is not needed. All the experiment is carried out in in vitro cell culture system.

3) Original western blot has given below. We have provided different exposure of blot PARP-1. Lower exposure blot is used in the final MS.

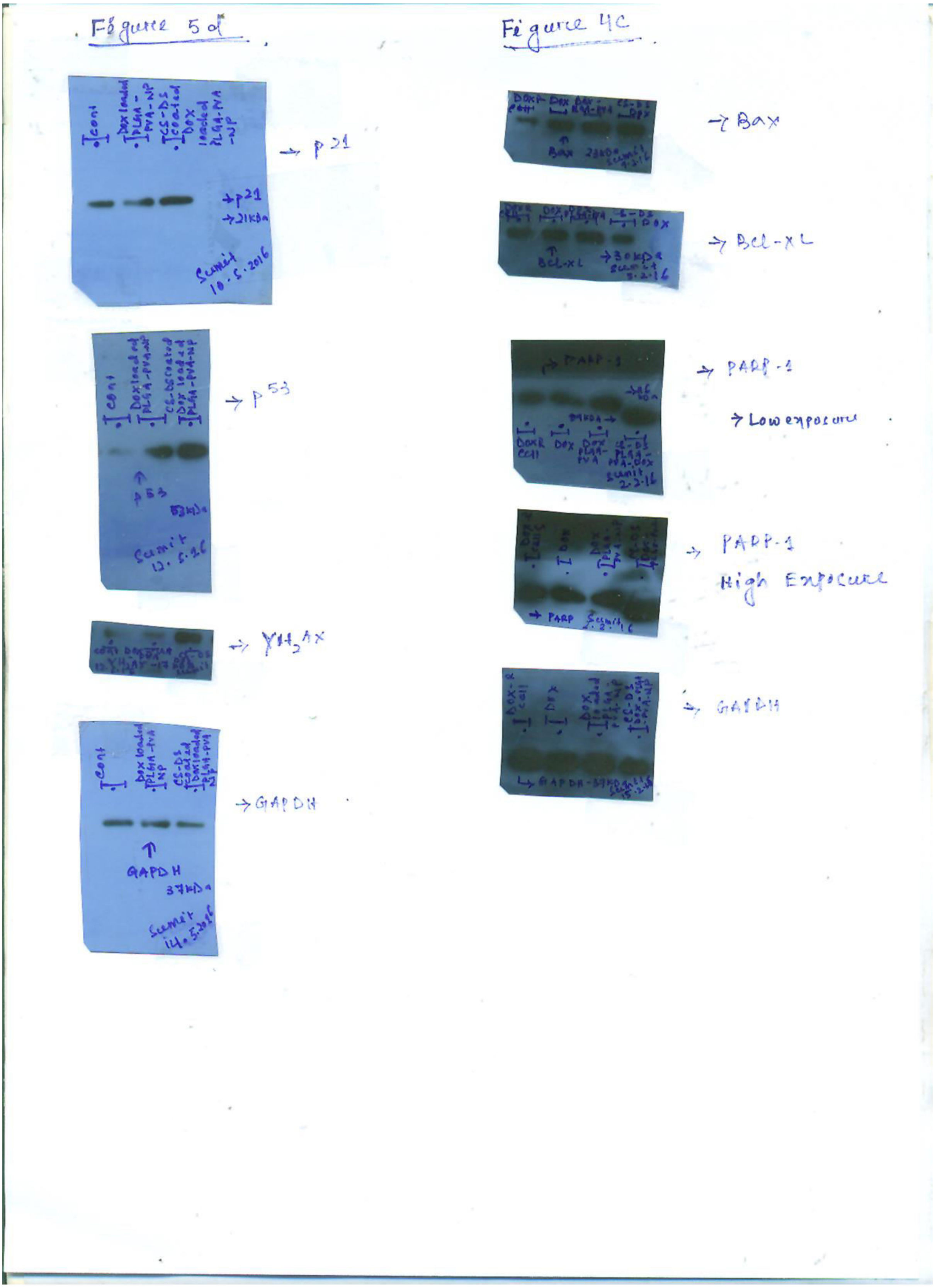

Figure: Origional western blot images.
